# Elucidating the Altered Transcriptional Programs in Breast Cancer using Independent Component Analysis

**DOI:** 10.1371/journal.pcbi.0030161

**Published:** 2007-08-17

**Authors:** Andrew E Teschendorff, Michel Journée, Pierre A Absil, Rodolphe Sepulchre, Carlos Caldas

**Affiliations:** 1 Breast Cancer Functional Genomics Laboratory, Cancer Research UK Cambridge Research Institute, Cambridge, United Kingdom; 2 Department of Oncology, University of Cambridge, Cambridge, United Kingdom; 3 Department of Electrical Engineering and Computer Science, University of Liège, Liège, Belgium; 4 Département d'Ingénierie Mathématique, Université Catholique de Louvain, Belgium; The University of Tokyo, Japan

## Abstract

The quantity of mRNA transcripts in a cell is determined by a complex interplay of cooperative and counteracting biological processes. Independent Component Analysis (ICA) is one of a few number of unsupervised algorithms that have been applied to microarray gene expression data in an attempt to understand phenotype differences in terms of changes in the activation/inhibition patterns of biological pathways. While the ICA model has been shown to outperform other linear representations of the data such as Principal Components Analysis (PCA), a validation using explicit pathway and regulatory element information has not yet been performed. We apply a range of popular ICA algorithms to six of the largest microarray cancer datasets and use pathway-knowledge and regulatory-element databases for validation. We show that ICA outperforms PCA and clustering-based methods in that ICA components map closer to known cancer-related pathways, regulatory modules, and cancer phenotypes. Furthermore, we identify cancer signalling and oncogenic pathways and regulatory modules that play a prominent role in breast cancer and relate the differential activation patterns of these to breast cancer phenotypes. Importantly, we find novel associations linking immune response and epithelial–mesenchymal transition pathways with estrogen receptor status and histological grade, respectively. In addition, we find associations linking the activity levels of biological pathways and transcription factors (NF1 and NFAT) with clinical outcome in breast cancer. ICA provides a framework for a more biologically relevant interpretation of genomewide transcriptomic data. Adopting ICA as the analysis tool of choice will help understand the phenotype–pathway relationship and thus help elucidate the molecular taxonomy of heterogeneous cancers and of other complex genetic diseases.

## Author Summary

The amount of a given transcript or protein in a cell is determined by a balance of expression and repression in a complex network of biological processes. This delicate balance is compromised in complex genetic diseases such as cancer by alterations in the activation patterns of functionally important biological processes known as pathways. Over the last years, a large number of microarray experiments profiling the expression levels of more than 20,000 human genes in hundreds of tumor samples have shown that most cancer types are heterogeneous diseases, each characterized by many different expression subtypes. The biological and clinical goal is to explain the observed tumor and clinical heterogeneity in terms of specific patterns of altered pathways. The bioinformatic challenge is therefore to devise mathematical tools that explicitly attempt to infer these altered pathways. To this end, we applied a signal processing tool in a meta-analysis of breast cancer, encompassing more than 800 tumor specimens derived from four different patient cohorts, and showed that this algorithm significantly outperforms popular standard bioinformatics tools in identifying altered pathways underlying breast cancer. These results show that the same tool could be applied to other complex human genetic diseases to better elucidate the underlying altered pathways.

## Introduction

Microarray technology is enabling genetic diseases like cancer to be studied in unprecedented detail, at both transcriptomic and genomic levels. A significant challenge that needs to be overcome to further our understanding of the relation between the quantitative transcriptome of a sample/cell and its phenotype is to unravel the complex mechanism that gives rise to the measured mRNA levels. The amount of a given mRNA transcript in a normal sample/cell is determined by a whole range of biological processes, some of which (e.g., transcription repression and degradation) act to reduce this number, while others (e.g., transcription factor induction) act to increase it. Therefore, it is natural to model the level of a given mRNA transcript as the net sum of a complex superposition of cooperating and counteracting biological processes, and, furthermore, to assume that disease is caused by aberrations in the activation patterns of these biological processes that upset the delicate balance between expression and repression in otherwise healthy tissue. Many distinct biological mechanisms that underlie the aberrations observed in human cancer have been identified, most notably copy-number changes [1] and epigenetic changes [2], yet it is the effect that these changes have downstream on the functional pathways that ultimately dictates whether these changes are pathological or not.

While several studies have recently characterised the altered functional pathways and transcriptional regulatory programs in human cancer, they have done so either by interrogating the expression data directly with previously characterised pathways, regulatory modules [3–6], and functionally related gene lists [7], or by interrogating derived “supervised” lists of genes for enrichment of biological function [8]. Hence, these studies have not attempted to *infer* the altered biological processes, which putatively map to alterations of known functional pathways and transcriptional regulatory programs. Thus, an unsupervised method that first infers the underlying altered biological processes and then relates these to aberrations in pathways or regulatory module activity levels is desirable.

A necessary property of such an algorithm is that it allows “gene-sharing,” so that a specific gene can be part of multiple distinct pathways. In this regard, it is worth noting that popular approaches for analysing transcriptomic data, such as hierarchical or k-means clustering, do not allow for genes to be shared by multiple biological processes, since they place a gene in a single cluster [9], and so they are not tailored to the problem of inferring altered pathways.

**Figure 1:**
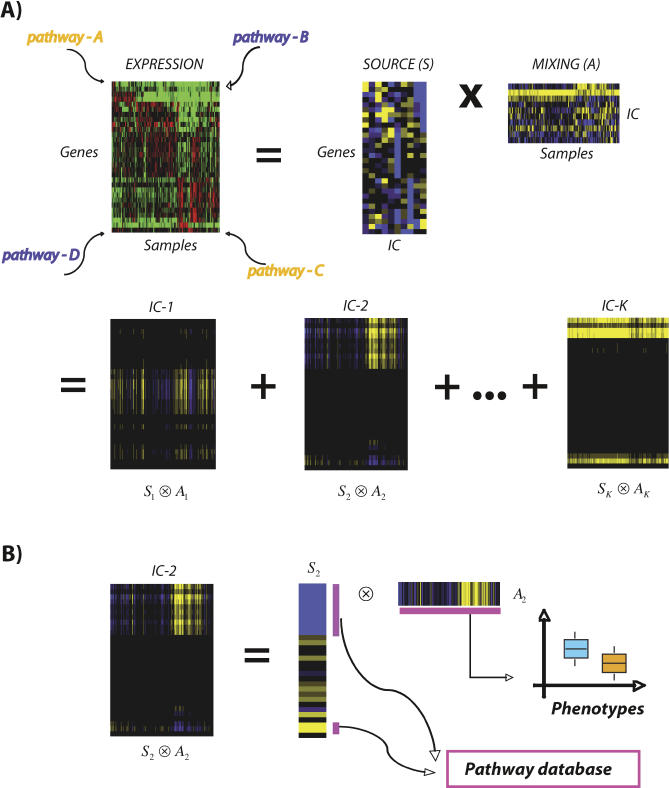
The ICA Model of Gene Expression Schematic depiction of the ICA model for gene expression. (A) Measured gene expression variations are caused by alterations in the activation levels of biological pathways. In the ICA model, the gene expression matrix is decomposed into the product of a “source” matrix *S* and a “mixing” matrix *A*, where *K* is the number of inferred independent components (IC) to which pathways and regulatory modules map. The columns of *S* describe the activation levels of genes in the various inferred independent components, while the rows of *A* give the activation levels of the independent components across tumor samples. The product of *S* and *A* can be written as a sum over the *IC* submatrices *IC*-1,*IC*-2,...*IC*-*K*. (B) *IC*–*k*–submatrix is obtained by multiplying the *k*-th column of *S*, *S_k_*, with the *k*-th row of *A, A_k_.* The genes with the largest absolute weights in *S_k_* are selected and tested for enrichment of biological pathways, while the distribution of weights in *A_k_* are tested for discriminatory power of phenotypes. (Colour codes for heatmaps: red, overexpression; green, underexpression; blue, upregulation; yellow, downregulation.)

Algorithms that allow genes to be part of multiple processes/clusters have also been extensively applied [10–12]. Among these, Singular Value Decomposition (SVD) or Principal Components Analysis (PCA) provides a linear representation of the data in terms of components that are *linearly* uncorrelated [12]. While this linear decorrelation of the data covariance matrix can uncover interesting biological information, it is also clear that it fails to map the components into independent biological processes, since there is no requirement for PCA components to be statistically independent. Mapping the data to independent biological processes, whereby independence is measured using a statistical criterion, should provide a more realistic representation of the data, since it explicitly recognises how the data was generated in the first place. This assumption, which is to be tested a posteriori, underlies the application of Independent Component Analysis (ICA) to gene expression data [13,14]. Specifically, ICA decomposes the expression data matrix *X* into a number of “components” (*k* = 1,2,..*K*), each of which is characterised by an activation pattern over genes (*S_k_*) and another over samples (*A_k_*) (Figure 1 and Materials and Methods),

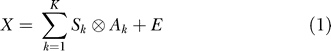
in such a way that the gene activation patterns (*S*_1_,*S*_2_,. . .,*S_K_*) are as statistically independent as possible while also minimising the residual “error” matrix *E* (in the above, ⊗ denotes the Kronecker tensor product). It is worth noting that while ICA also provides a linear decomposition of the data matrix, the requirement of statistical independence implies that the data covariance matrix is decorrelated in a *non-linear* fashion, in contrast to PCA where the decorrelation is performed linearly.

Many studies have shown the value of ICA in the gene expression context as a dimensional reduction and gene-functional discovery tool [15–20] and also as a potential tool for classification and diagnosis [21,22]. To validate the ICA model, most of these studies used the Gene Ontology (GO) framework [23]. However, GO does not provide the best framework in which to evaluate the ICA paradigm, since many genes with the same GO term annotation may not be part of the same biological pathway or may not be under the control of the same regulatory motif, and vice versa. In fact, to date no study has evaluated the ICA paradigm in the explicit context of biological pathways and regulatory modules.

In this work we apply various popular ICA algorithms to six of the largest available microarray cancer datasets. We focus on breast cancer for two reasons. First, for this type of cancer many large patient cohorts that have been profiled with microarrays are available. Second, breast cancer is a highly heterogeneous disease and hence it provides a more challenging (and hence suitable) arena in which to compare and evaluate different methodologies. We also use two large microarray datasets from two other cancer types to show that our results are valid more generally. The aim of our work is 2-fold. First, to test the ICA paradigm by showing that it significantly outperforms both a gene-sharing method that does not use the statistical independence criterion (PCA) and a traditional (“non–gene-sharing”) clustering method (k-means). We achieve this by using a pathway and regulatory module–based framework for validation. The second aim is to find the most frequently altered pathways and regulatory modules in human breast cancer and to explore their relationship to breast cancer phenotypes.

## Results

### Testing the ICA Paradigm

The main modelling hypothesis underlying the application of ICA to gene expression data is that the expression level of a gene is determined by a linear superposition of biological processes, some of which try to express it, while other contending processes try to suppress it (Figure 1). It is assumed that these biological processes correspond to activation or inhibition of single pathways or sets of highly correlated pathways, and that each of these pathways only affects a relatively small percentage of all genes. Because of the statistical independence assumption inherent in the ICA inference process, we would expect the identified independent components to map more closely to known pathways than an alternative linear decomposition method, like PCA, that does not use the statistical independence criterion. Similarly, we would expect ICA components to map closer to pathways than clusters derived from popular clustering algorithms such as k-means or hierarchical clustering.

To test the modeling hypothesis of ICA for expression data, we first asked how well the inferred components mapped to known pathways, as curated in the MSigDB pathway database [24] (Materials and Methods, Table S1). This strategy was initially applied to a total of six breast cancer microarray datasets (“Perou” [25], “JRH-1” [26], “Vijver” [27], “Wang” [28], “Naderi” [29], “JRH-2” [30]), summarised in Table 1, and for four different implementations of the ICA algorithm (“fastICA”, “JointDiag”, “KernelICA”, and “Radical”) [31–34] as well as for ordinary PCA and two versions of k-means clustering (PCA-KM and MVG-KM) (Materials and Methods and Protocol S1). For each of the ICA algorithms and PCA, we inferred ten components and selected the genes based on their weights in the corresponding column of the source matrix *S* (Materials and Methods). The average number of genes selected per component ranged from 50 to 200 depending on the cohort (Table S2). For the two k-means clustering algorithms, ten gene clusters were inferred on subsets of most variable genes to ensure that the average number of genes per cluster was similar to that of the PCA and ICA components. This step was necessary to ensure an objective comparison of the different algorithms. In what follows we also use the term component to denote clusters. To evaluate how close the inferred components of a given algorithm in a particular cohort mapped to existing pathways, we defined a pathway enrichment index, PEI, as follows. For each component *i* and pathway *p,* we first evaluated the significance of enrichment of genes in that pathway in the selected feature set of the component by using the hypergeometric test (see Materials and Methods). This yielded for each component *i* and pathway *p* a *p*-value *P_ip_*. Correction for multiple testing was done using the Benjamini-Hochberg procedure to obtain an estimate for the false discovery rate (FDR). A component *i* was then declared enriched for a pathway *p* if the Benjamini-Hochberg corrected *p*-value was less than 0.05. Hence, we would expect approximately 5% of significant tests to be false positives. Finally, we counted the number of pathways enriched in at least one component and defined the PEI as the corresponding fraction of enriched pathways.

**Table 1:**
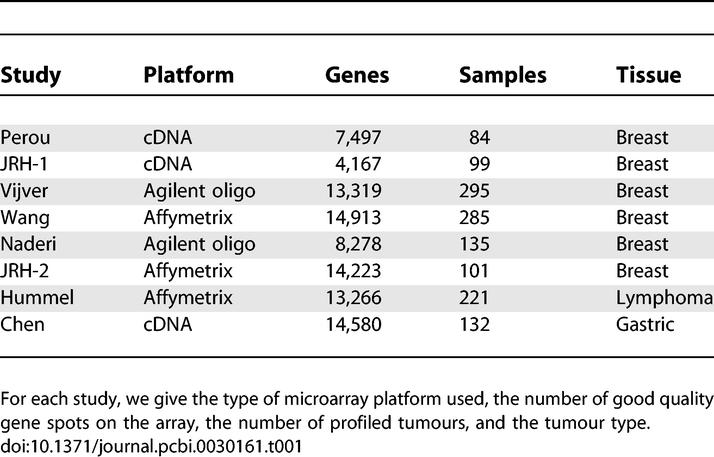
Breast Cancer Cohorts

### ICA Components Map Closer to Known Biological Pathways

**Figure 2:**
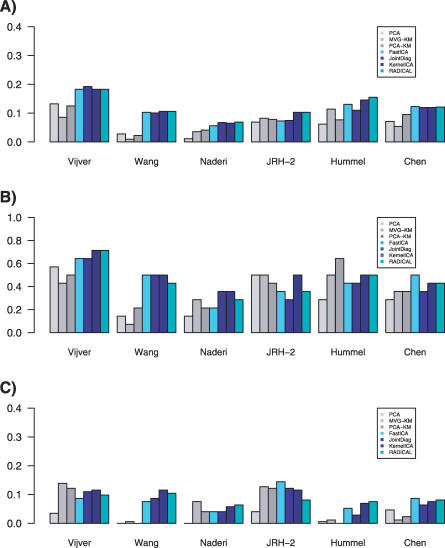
Testing the ICA Paradigm (A) For each cohort and method, we give the pathway enrichment index, PEI, defined by the fraction of biological pathways (536 in total) found enriched in at least one component. (B) For each cohort and method, we give the fraction of cancer-signalling and oncogenic pathways (14 in total) successfully mapped by the inferred components. (C) For each cohort and method, we give the fraction of motif-regulatory gene sets (173 in total) captured by the inferred components.

The PEI for each of the seven methods (“PCA”, “MVG-KM”, “PCA-KM”, “fastICA”, “JointDiag”, “KernelICA”, “Radical”, and “PCA”) and the four largest breast cancer sets (“Vijver”, “Wang”, “Naderi”, “JRH-2”) are shown in Figure 2A (the results for all six breast cancer cohorts are presented in Figure S1). This showed that across the four major cohorts the PEI was higher for ICA algorithms when compared with PCA and the clustering-based methods. Interestingly, for the two largest cohorts (“Vijver” and “Wang”), the degree of improvement in the PEI of ICA over PCA, MVG-KM, and PCA-KM was highest. In contrast, for the smaller cohorts (e.g., “Perou” and “JRH-1”), the degree of improvement of ICA over PCA or KM was less marked. Hence, since we found that cohort size had a significant impact on the inferred components, we restricted all subsequent analysis to the four major breast cancer cohorts. It is also noteworthy that when comparing the various ICA algorithms with each other we didn't observe any appreciable difference in their respective PEI.

### ICA Captures More Cancer Signalling and Oncogenic Pathways in Breast Cancer

To investigate this further, we next compared the algorithms on the subset of nine cancer-signalling pathways from the curated resource NETPATH (http://www.netpath.org) and five oncogenic pathways [35]. These are pathways that are frequently altered in cancer and hence we would expect many of these to be captured by the ICA algorithm. Thus, for each method and study we counted the number of pathways that were enriched in any of the components (Figure 2B). This showed that in the three largest breast cancer studies (“Vijver”, “Wang”, and “Naderi”), PCA and the KM-methods captured the least number of pathways. In the two largest cohorts (“Vijver” and “Wang”), for example, the “RADICAL” ICA algorithm captured ten and six of the 14 pathways, while PCA captured eight and two pathways, respectively.

### ICA-Derived Components Map Closer to Regulatory Modules

As a further validation that ICA outperforms PCA, we investigated the relation of the derived components with regulatory modules. Specifically, we tested the selected gene sets from each component for enrichment of genes with common regulatory motifs in their promoters and 3′ UTRs [36]. Under the ICA paradigm we would expect genes that are under the common regulatory control of a transcription factor to appear in the same ICA component. Thus, for each breast cancer cohort and method we counted the number of regulatory motifs whose associated genes were overrepresented in components (Figure 2C), using as before the hypergeometric test to test for significant enrichment (Materials and Methods). This showed that PCA performed worst out of all algorithms. In two cohorts (“Wang” and “Naderi”), none of the PCA components was associated with any of the 173 distinct regulatory motifs. In contrast, the components derived by ICA algorithms were consistently associated with regulatory motifs. Interestingly, the improvement of ICA over KM-based methods was less marked with only study (“Wang”) showing a substantial improvement (Figure 2C).

### ICA Outperforms PCA and KM-Clustering across Different Cancer Types

The results above show that ICA provided a more biologically meaningful decomposition of breast cancer expression data than PCA or KM-based methods. This suggested to us that similar results would hold in other types of cancer. To check this, we analysed two additional large microarray datasets, one profiling 221 lymphomas [37] (“Hummel”) and another profiling 132 gastric cancers [38] (“Chen”) (see Table 1). The same analysis on these two additional datasets confirmed that the PEI was higher for ICA when compared with PCA or KM-clustering methods (Figure 2A), and that ICA components also mapped closer to known regulatory motifs (Figure 2C).

### ICA Provides a More Robust Identification of Differentially Activated Biological Pathways and Regulatory Modules in Breast Cancer

**Figure 3:**
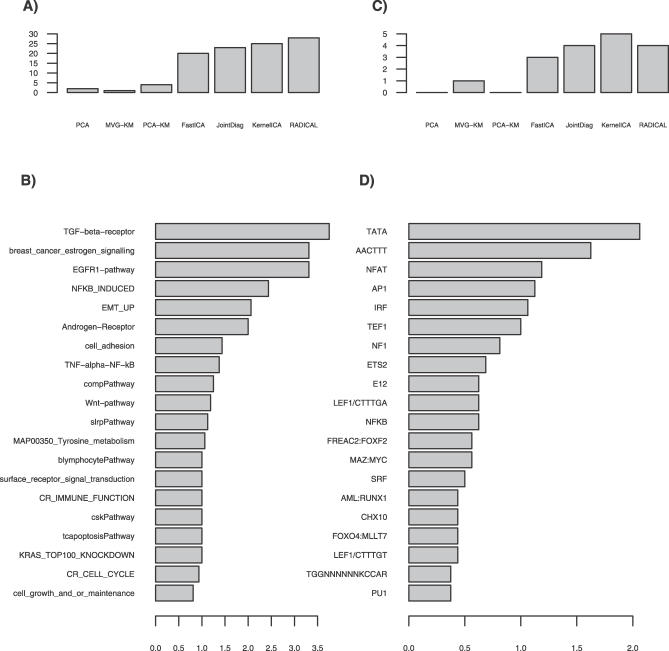
Most Consistent and Frequently Mapped Pathways and Regulatory Motifs (A) For each method, we compare the number of pathways that were consistently mapped to components across the four major breast cancer studies. (B) Twenty of the most frequently mapped pathways by ICA. The scores give the average number of ICA components in which the pathway was mapped. (C) For each method, we give the number of motif-regulatory gene sets consistently mapped to components across the four major breast cancer cohorts. (D) The 20 most frequently mapped transcription factors/regulatory motifs by ICA. The scores give the average number of ICA components in which the regulatory module of the motif was mapped.

To investigate the robustness of the algorithms, we next compared the ability of the algorithms to identify pathways and regulatory modules that were differentially activated independent of the breast cancer cohort used. Two important observations that were independent of the ICA algorithm and cohort used could be derived from the heatmaps of differential activation of pathways and regulatory modules (Figures S2–S5). First, ICA identified many more pathways that were consistently differentially activated across all four breast cancer cohorts (Figure 3A). This further confirmed that the associations between components and pathways as picked out by ICA were more robust and consistent between cohorts than those identified through PCA, MVG-KM, or PCA-KM. Among the pathways that were found to map most frequently and consistently to components were those related to estrogen signalling as well as to other important breast cancer–signalling pathways such as the EGFR1 and TGF-β pathways (Figures 3B and S2–S5). We also found cell-adhesion, immune-response, cell-cycle, and metabolic pathways to be commonly differentially activated across the cohorts. While breast cancer studies have found study-specific gene clusters associated with cell-cycle, estrogen-response, cell-adhesion, and immune-response functions, our results show that expression variation across breast tumours can be understood in terms of single pathways (i.e., a fixed common set of genes for all studies) that relate to these biological functions.

Second, we also observed that ICA outperformed PCA, MVG-KM, and PCA-KM in identifying regulatory modules that were consistently differentially activated across cohorts (Figure 3C). Specifically, the KernelICA algorithm identified the regulatory modules TATA, AACTTT, NFAT, IRF, and NF1, while MVG-KM only picked out TATA, with PCA and PCA-KM failing to capture any regulatory module. Among the motifs with regulatory gene modules that were most frequently captured by independent components, we found several with important general (e.g., TATA) and specific transcription factors (e.g., NF1 and ETS2) (Figures 3D and S2–S5).

### Differentially Activated Pathways and Regulatory Modules Associate with Breast Cancer Phenotypes

We next asked whether components mapping into the various pathways/modules were associated with breast cancer phenotypes. Specifically, we considered three categorical phenotypes: estrogen receptor (ER) status (0,1), histological grade (1,2,3), and outcome (0,1). To evaluate statistical significance of any association between a component *k* and phenotype, we considered the distribution of weights from the corresponding row of the mixing matrix, i.e., *A_k_* (Materials and Methods), across the different categories. We used the Wilcoxon rank-sum test for the two binary phenotypes and the Kruskal-Wallis test for histological grade. Because of the clustering nature of the MVG-KM and PCA-KM algorithms, in these two cases we first applied k-means over the genes in the cluster to partition the samples into two groups and subsequently used Fisher's exact test to determine whether the phenotype distribution across the two groups was significantly different from random or not.

This revealed a complex pattern of significant associations with several components differentiating breast tumours according to ER status and histological grade (Figures S2–S5). It is notable that in all cohorts ICA components associating with clinical outcome were also found, while PCA generally did not. Another feature was the fact that more and stronger phenotype associations were uncovered by using ICA as compared with PCA. On the other hand, MVG-KM performed as well as ICA in mapping to ER, grade, and outcome phenotypes.

**Figure 4:**
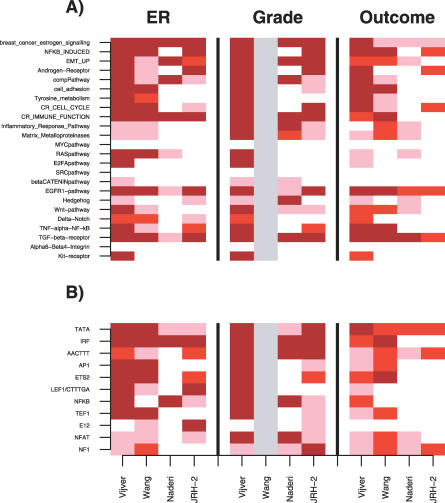
Heatmaps of Association of Pathways and Regulatory Modules with Breast Cancer Phenotypes For three phenotypes (ER, Grade, Outcome), we show heatmaps of association between phenotypes and selected pathways (A) and selected regulatory motifs (B), as revealed by the four ICA algorithms across the four major breast cancer cohorts. For phenotypes, we used a *p*-value threshold of 0.05 to establish whether an ICA component was associated with that phenotype. For pathways and regulatory modules, we used the Benjamini corrected *p*-values as before. For each cohort, we then counted the number of ICA algorithms that found a component linking a phenotype with a pathway/regulatory module, which was colour-coded as 4 (dark red), 3 (red), 2 or 1 (pink), and 0 (white). For Wang's cohort, grade information was unavailable and is colour-coded as grey.

**Figure 5:**
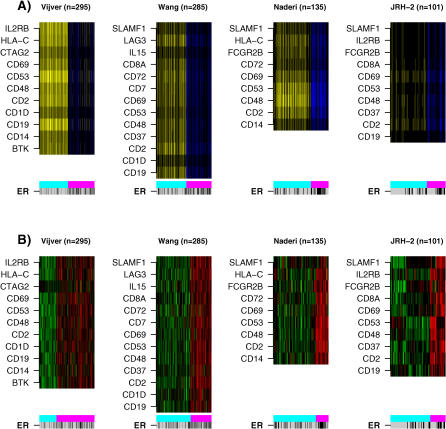
The Association of Immune Response with Estrogen Receptor Status (A) For each major breast cancer cohort, we give the heatmap of component expression values for the component enriched for the immune-response pathway characterised in [39]. Thus, the heatmap matrix shown is *S_gk_A_ks_* where *k* is the component enriched for the immune response pathway, *g* is any gene found on the array that is also in the pathway and the selected feature set of the component, and *s* denotes the tumour sample. Samples have been ordered according to a k-means (k = 2) clustering over the set of genes. The ICA algorithm for which this heatmap is shown is the KernelICA algorithm. Blue denotes “upregulation,” yellow “downregulation.” For the samples, black denotes an ER− and grey an ER + tumour. (B) For each major breast cancer cohort, we give the heatmap of expression values for the same set of genes as in (A). Thus, the heatmap matrix shown is *X_gs_* where *X_gs_* denotes the measured expression level of gene *g* in sample *s*. As before, samples have been ordered according to a k-means (k = 2) clustering over the represented genes. Red denotes relative overexpression, green underexpression. Magenta denotes the upregulated cluster, cyan the downregulated cluster.

**Figure 6:**
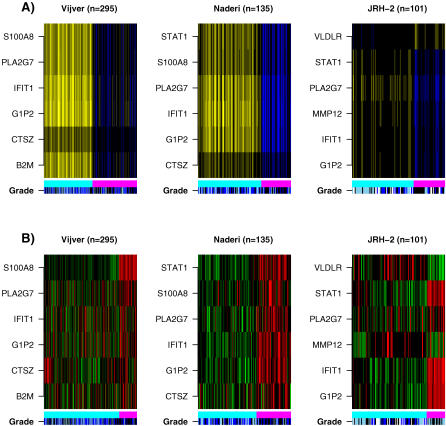
The Association of Epithelial–Mesenchymal Transition with Histological Grade (A) For each major breast cancer cohort where grade information was available, we give the heatmap of component expression values for the component enriched for the EMT pathway characterised in [41]. Thus, the heatmap matrix shown is *S_gk_A_ks_* where *k* is the component enriched for the EMT pathway, *g* is any gene found on the array that is also in the pathway and the selected feature set of the component, and *s* denotes the tumour sample. The ICA algorithm for which this heatmap is shown is the KernelICA algorithm. Samples have been ordered according to a k-means (k = 2) clustering over the set of genes. Blue denotes “upregulation,” yellow “downregulation.” For the samples, histological grade is colour-coded as black (high-grade), blue (intermediate grade), and skyblue (low-grade). (B) For each major breast cancer cohort, we give the heatmap of expression values for the same set of genes as in (A). Thus, the heatmap matrix shown is *X_gs_* where *X_gs_* denotes the measured expression level of gene *g* in sample *s*. As before, samples have been ordered according to a hierarchical clustering over the represented genes. Red denotes relative overexpression, green underexpression. Magenta denotes the upregulated cluster, cyan the downregulated cluster.

Since we characterised each component in terms of the differential activation pattern of cancer-related pathways and regulatory modules, for those components associated with a phenotype we were able to link the corresponding pathways and regulatory motifs with the phenotype (Figure 4). This led to several well-known but also novel observations. First, as expected, ICA components that were strongly associated with ER status were frequently mapped to the estrogen signalling pathway. Second, ICA components that mapped to the CR (cancer related) cell-cycle pathway [39] were frequently associated with either grade or outcome. The association between cell-cycle genes and grade or outcome is well-known [26,30,40], and our finding further shows that an independently characterised cell-cycle pathway associates with these clinical variables across multiple studies. Third, we observed that pathways relating to immune response functions and the classical complement pathway were frequently correlated with ER status, grade, and, although less frequently, with clinical outcome. For example, we found in each of the four major breast cancer cohorts an ICA component that mapped to the CR immune response pathway [39], and which was consistently overactivated in ER− relative to ER+ tumours (Figure 5A and Table 2). We note that the same set of genes, when viewed over the measured expression matrix also separated the samples according to ER status (Figure 5B and Table 2). Fourth, in all studies where grade information was available, an ICA component mapping to either matrix-metalloproteinases (MMP) or the cell-adhesion pathway was found to be associated with histological grade. In three studies (“Wang”, “Vijver”, and “Naderi”), the MMP pathway was also found to be associated with outcome. Another interesting pathway we found to be associated with histological grade was an epithelial–mesenchymal transition (EMT) signalling pathway characterised in [41]. Specifically, ICA revealed a component driving upregulation of genes involved in EMT in poorly differentiated tumours relative to low-grade tumours across the three studies where grade information was available (Figure 6A and Table 3). When the same set of genes defining the EMT pathway was viewed over the measured expression matrix, their pathway coherence was less evident, although the association with grade was still revealed by k-means clustering (Figure 6B and Table 3).

**Table 2:**
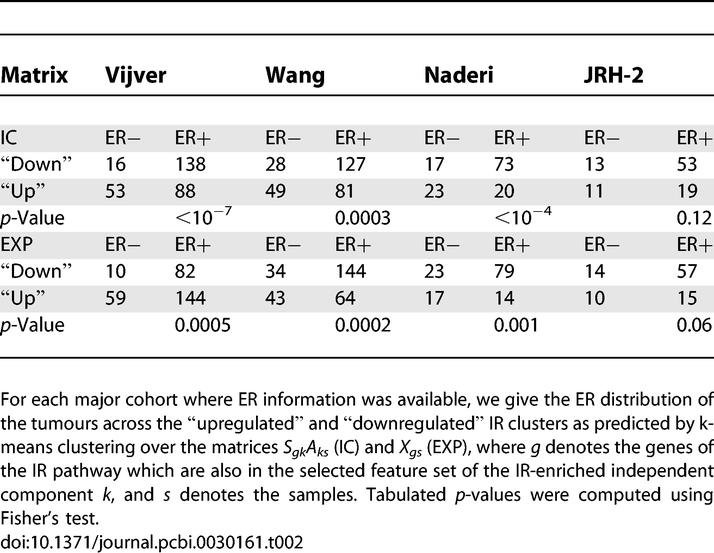
Association of Immune Response with Estrogen Receptor Status

**Table 3:**
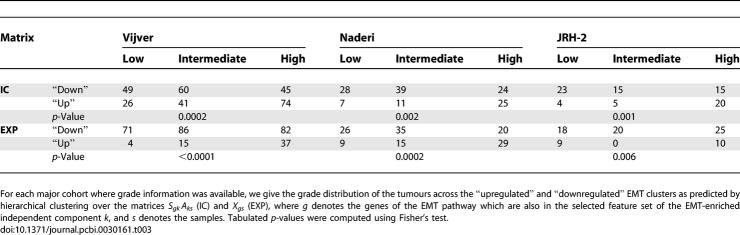
Association of Epithelial–Mesenthymal Transition with Grade

The parallel analysis for regulatory motifs and breast cancer phenotypes provided direct links between the associated transcription factors and clinical variables (Figure 4B). Strikingly, we found that the interferon regulatory factor (IRF) showed the strongest associations with both the ER and grade phenotypes. The regulatory module associated with the TATA box was also frequently associated with ER, grade, and outcome. Interestingly, we found differential activation of the regulatory modules associated with the neurofibromin-1 (NF1), NFAT, and ETS2 transcription factors to be associated with clinical outcome, which is significant in view of the results of several recent studies linking these transcription factors with the metastatic and cell-growth properties of breast cancer cells [42–46].

**Figure 7:**
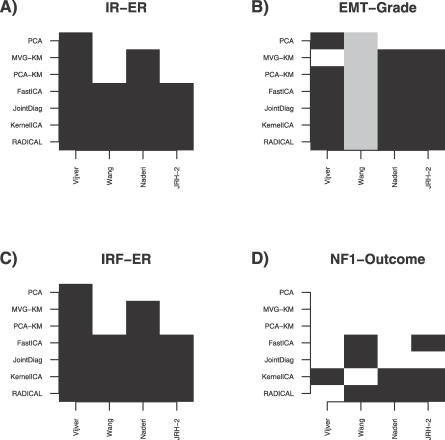
Inter-Method Comparison of Selected Associations of Pathways and Regulatory Modules with Breast Cancer Phenotypes The ability of the various methods to capture novel biological associations between pathways/regulatory modules and phenotypes is represented as a binary heatmap across methods and cohorts. (A) Immune response pathway and ER status, (B) EMT-pathway and grade, (C) IRF and ER status, (D) Neurofibromin-1 and clinical outcome. Black denotes a statistically significant association between a pathway/regulatory module and the phenotype in question, white means no evidence of an association.

It is important to point out that ICA facilitated the identification of many of the biological associations in comparison with PCA, MVG-KM, and PCA-KM (Figure 7). Thus, for example, we can see that the association between immune response and ER status was found in all cohorts by any one of the four ICA algorithms, whereas PCA and the KM methods were generally not as robust (Figure 7A). A similar observation could be made for the associations between the EMT pathway and grade, and that of the IRF module and ER status (Figure 7B and 7C). For the case of NF1 and clinical outcome, this association was not identified by PCA or the KM-based methods (Figure 7D).

Finally, we verified that in many cases the identified associations were independent, in the sense that the component(s) or genes linking a pathway with a phenotype could be different from the one(s) linking another pathway with the same phenotype. For example, we noted that this was the case for the associations of the cell-adhesion and estrogen-signalling pathways with grade (see Figures S2 and S4). Similarly, the associations of the immune response pathway and IRF module with ER status (Figure 7A and 7C) could not be attributed to a common gene subset selection, since the pathway and module gene sets shared no genes in common.

### Association Networks

**Figure 8:**
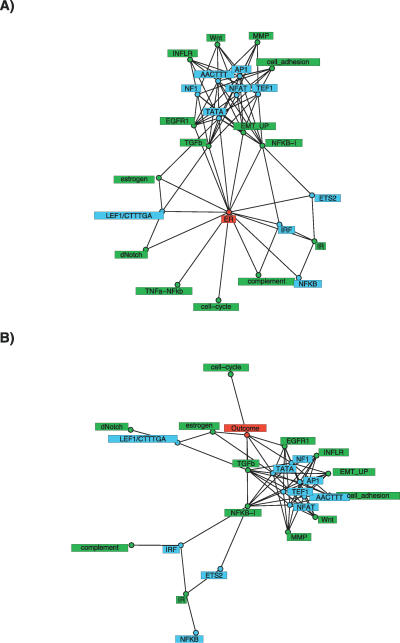
Association Networks Average association networks shown for ER status (A) and clinical outcome (B). Only edges between phenotypes, pathways, and transcription factors are shown (for the sake of clarity, edges between any two pathways, transcription factors, or phenotypes are not shown). An edge between two nodes was defined if the association between the two nodes was present in at least three out of the four studies, as predicted by the KernelICA algorithm. The diagrams are colour-coded as follows: phenotype (red), pathways (green), and transcription factors/binding motifs (blue). INFLR, inflammatory response; TM, tyrosine metabolism.

Networks are a useful tool for graphically representing relational structures between many layers of organisation. In our application, we sought to construct a network of associations, linking breast cancer phenotypes, pathways, and regulatory modules with each other as the nodes in the network. To represent only the most salient and robust features, we focused attention on those pathways and regulatory modules with most phenotypic associations (Figure 4) and on those associations that were most consistently predicted across cohorts. Thus, we constructed an average network over the networks for each study by defining a link between any two nodes in the network if there were at least three studies in which there was a link between the two nodes, as predicted by ICA (Figure 8) (KernelICA was used but the other ICA algorithms gave similar networks). This revealed a complex network of associations between transcription factors, pathways, and breast cancer phenotypes. Strengthening the association of immune response with ER status further, we found triangular relationships involving the NF-κβ, ETS2, and IRF transcription factors (Figure 8A), which is plausible in view of their role in regulating immune response pathways [47–49]. The corresponding network for clinical outcome showed that apart from the cell-cycle and estrogen-signalling pathways, only the EGFR1 and TGF-β pathways were consistently associated with outcome (Figure 8B).

## Discussion

In our view, it is most natural to analyse gene expression data in the context of a generative model, however approximate this model is to the true underlying mechanism that gives rise to the measured expression levels. ICA provides such a generative model since it explicitly recognises how the data was generated in the first place. By comparing ICA with PCA and clustering-based methods, we have shown that a more realistic representation of the data is obtained by allowing “gene-sharing” and using the statistical independence criterion (non-linear decorrelation) in the inference process (ICA), as opposed to not allowing gene-sharing (MVG-KM, PCA-KM) and only using a linear decorrelation criterion (PCA). We showed this on a total of six cancer microarray datasets, using existing pathway knowledge and gene regulatory module databases for evaluation. Specifically, we found that ICA components mapped closer to cancer-related pathways as well as to gene modules that are under the control of a common regulatory motif. It is worth pointing out though that the improvement of ICA over KM methods was less marked in the case of regulatory motifs, as we would expect, since a clustering method is partially tailored to finding co-regulatory structure. Importantly, when comparing the results across cohorts, we found that ICA algorithms were much more robust than PCA or KM-based methods, in the sense that pathways that were found to be differentially activated through ICA in one cohort were also consistently differentially activated in the other cohorts. A similar observation could also be made for the regulatory motifs and their regulatees. For example, using PCA or PCA-KM, no regulatory module was found to be differentially activated across all four major breast cancer studies, while the ICA algorithms found an average of four modules. The most likely explanation for the relatively smaller number of regulatory modules found in common across the four studies, as compared with pathways, is that many regulatory modules important to breast cancer have yet to be elucidated.

Of note, we also performed the enrichment analysis of the independent components for chromosomal bands (using the MSigDB database), which confirmed that the independent components were not capturing transcriptional programs localised to specific chromosomal regions. Instead, we believe that the inferred independent components encapsulate “net” transcriptional programs that act globally and downstream of the epigenetic and genetic modifications underlying cancer.

We also found that ICA components were associated more often with known breast cancer phenotypes, including clinical outcome, and that these associations were also much stronger for ICA than for PCA. While this result is to be expected, since ICA components map closer to pathways that have been characterised using phenotypic information, one should also bear in mind that these pathways were derived from independent experiments; hence, the stronger associations between components, pathways, and phenotypes as revealed by ICA provides a validation, not only of the algorithm itself, but also of the characterised pathways.

Another important observation was the presence of multiple components showing an association with a particular pathway, regulatory module, or phenotype. This suggests that a significant proportion of pathways are part of multiple biological processes. Alternatively, the presence of multiple components enriched for a given pathway may reflect distinct gene subset selection, which in turn suggests that the pathways in MSigDB and NETPATH may need to be refined further. In the context of phenotypes, the presence of multiple components correlating with ER status, grade, or outcome, is suggestive of tumour heterogeneity, since, more often than not, the differential distribution of the phenotype across samples is dependent on the precise component. Hence, the fingerprint patterns of pathway activation derived from ICA could potentially form the basis for further clinically relevant definitions of breast cancer subtypes.

In an exploratory analysis, ICA revealed many interesting associations between pathways and phenotypes that can form the basis for future investigations. While all methods were able to identify the expected relationships of the estrogen-signalling pathway with ER status and cell-cycle pathway with histological grade, ICA clearly outperformed PCA and KM-clustering in identifying many other biologically relevant associations (Figure 7). For example, ICA consistently found an expression mode involving immune response pathways that was upregulated in ER− versus ER+ tumours. Thus, while the relation between immune response and ER status is still poorly understood [50], our results clearly point at an important link between the immune response and estrogen signalling in breast cancer, which needs to be explored further. ICA also revealed interesting associations of the EMT-signalling, cell-adhesion, and MMP pathways with histological grade and clinical outcome. Specifically, we found a component upregulating EMT genes in high-grade versus low-grade tumours, and which was statistically significant in three major cohorts. The association between the activity level of the cell-adhesion and MMP pathways with clinical outcome as revealed by ICA is also noteworthy given that supervised approaches tend to only find genes related to cell-cycle pathways, as these are the strongest predictors of grade and outcome. While the association of cell-adhesion genes with outcome has been noted before in breast cancer [29] and to a lesser extent in gastric cancer [51], here we show that this result holds for a specific pathway and across several breast cancer cohorts. ICA, in contrast to PCA and KM-clustering, also identified interesting associations between transcription factor modules and phenotypes (Figure 7). For instance, it found strong associations between the IRF and ER status and between NF1 and clinical outcome, as well as an association between NFAT and outcome (Figure 4). These associations are plausible given that changes in NFAT have been shown to alter the metastatic and growth properties of breast cancer cells [42–44], and given the important role NF1 and IRF play in breast cancer generally [52–56].

It could be argued that both IR- and cell-adhesion pathways are differentially activated across tumours merely as a result of lymphocytic or stromal contamination, respectively. However, microarray studies profiling breast cancer cell lines (BCL) have shown that genes associated with IR- and cell-adhesion functions are also differentially regulated across cell lines [25,57]. In particular, it was shown that genes related to cell-adhesion functions were overexpressed in ER− compared with ER+ cell-lines [57]. While the study in [57] did not explicitly mention the differential expression of immune response genes, we verified, by applying ICA to this set of only 31 breast cancer cell lines (BCL), that an independent component enriched for immune response genes was present and that it correlated with the ER status of the cell lines (Figure S6). This provided further validation of the link between differential regulation of immune response pathways with the ER status of breast cancer cells, while also simultaneously confirming that the differential regulation of these genes across the tumour set is not necessarily related to varying degrees of lymphocytic infiltration.

Generally, we found that genes selected in the same independent component showed a relatively strong co-expression pattern (Figure 5B). It follows that ICA components can often be given a biological interpretation similar to that of clusters inferred through, say, hierarchical or k-means clustering. To illustrate this with another example, we considered the case of estrogen signalling and ER status. This showed that clustering over the genes selected in an IC that was associated with estrogen signalling and ER status yielded similar heatmaps for the measured expression matrix and the IC submatrix, and, furthermore, for both heatmaps the association with the phenotype was evident (Figure S7).

On the other hand, ICA also found “non-trivial” associations, such as the association of the EMT pathway with grade (Figure 6A), where the functional relationship of the genes in the same pathway was not as evident from the gene expression matrix (Figure 6B). Given that genes are shared by multiple pathways, the functional relationship of the genes may indeed not manifest itself as a strong co-expression pattern. Thus, it would appear that ICA, through the statistical independence criterion, which effectively uses non-linear correlation measures (as opposed to mere linear co-expression) to determine common functionality, is able to capture non-trivial functional relationships of genes in a common pathway, in spite of the fact that these genes may not exhibit strong co-expression.

In summary, this work is the first to our knowledge to validate the ICA paradigm using a framework based on existing pathway-knowledge and regulatory-module databases. Moreover, it confirms the added value of ICA over PCA and clustering-based methods in identifying novel associations of known pathways and regulatory modules with breast cancer phenotypes. Our results also indicate that larger datasets may be required before a more complete understanding of the ICA model in the gene expression context can be obtained, as well as to understand to what degree ICA can help in defining a more clinically relevant molecular taxonomy of breast cancer.

## Materials and Methods

### A pathway-knowledge database.

To test the ICA model, we first generated a comprehensive list of pathways, most of which are known to be directly or indirectly involved in cancer biology. To compile this list, we used the Molecular Signatures Database MSigDB [24], which included 522 distinct pathways curated from the literature and from other databases such as KEGG (http://www.genome.jp/kegg/) and CGAP (http://cgap.nci.nih.gov/). We augmented this list with known oncogenic pathways recently derived in [35] and cancer-signalling pathways from NETPATH (http://www.netpath.org), yielding a total of 536 pathways. Not all of these pathways had sufficient representation across the six major studies. Specifically, out of these 536 pathways, 277 had at least five genes represented on each of the six microarray platforms (probes on specific microarrays were also filtered based on quality, which explains why there wasn't a higher percentage of pathway gene lists with sufficient representation). The full list of pathways used are summarised in Table S1 in terms of their representation on each of the arrays.

### Regulatory motifs.

We used the sequence-derived regulatory motifs in human promoters and 3′ UTRs from [36]. For each such motif we defined the associated regulatory gene module as the set of genes having this motif in their promoters or 3′ UTR, as provided in MSigDB [24]. The selected feature sets of the inferred components were tested for enrichment of regulatory modules, which provided us with putative links between components and the transcription factors that bind to these motifs.

### The ICA and PCA Models.

Briefly, we review the ICA model [58] as used in this work. Let *X_gs_* denote the normalised data matrix of expression values where *g* = 1,. . .,*n* denotes the genes and *s* = 1,. . . , *N* denotes the samples. We assume further that *X* has been normalised so that the mean of each column of *X* is zero. Then ICA (or PCA) produces an approximate decomposition of the matrix *X* into the product of two matrices *S* (the “source” matrix) and *A* (the “mixing” matrix):

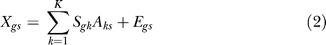
where *K* ≤ *min*{*n*,*N*} is the number of components to be computed. When *K* is strictly smaller than *min*{*n*,*N*}, it is in general impossible to pick *S* and *A* such that the error matrix vanishes. Therefore, the algorithms aim at making *E* as small as possible, usually in the least squares sense. This condition on *E* still leaves much leeway to select the matrices *S* and *A*.

PCA consists of identifying an orthonormal matrix *S* (i.e., 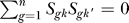 for all *k* ≠ *k*′, and 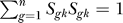 for all *k*) and an orthogonal matrix *A* (i.e., 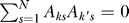 for all *k* ≠ *k*′) so that the data covariance matrix is diagonalised. In comparison, most ICA algorithms start with a preprocessing step, in which the means of the columns of *X* are set to zero, followed by a PCA. Thus, as with PCA itself, this first requires an orthonormal matrix *S*′ and an orthogonal matrix *A*′ such that *X* = *S*′*A*′ + *E*′. It should be noted that orthonormality of *S*′ implies a sample covariance between the columns of *S*′ that equals zero. The ICA step per se amounts to then finding a transformation *W* of *S*′,

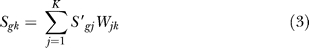
such that the columns of *S* are “as independent as possible”. Most ICA methods consider that the zero covariance property of *S*′ is compatible with this goal, hence they preserve this property in *S*′ by restricting *W* to the set of *K* × *K* orthogonal transformations. The ICA algorithms, thus, search for an orthogonal matrix *W* that maximises the statistical independence of the columns of *S*′. The mixing matrix finally equals

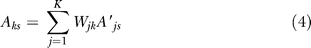
and the error *E* is identical to *E*′.

A quantitative measure of independence between measurements of random variables, in this case the columns of *S*′, is provided by a contrast function. The only requirement on the contrast function is that it goes with probability one to a prescribed extremum (usually zero) if and only if the random variables are statistically independent and as the number of measurements *n* goes to infinity. This leaves many possibilities for the contrast function, leading to a variety of ICA algorithms, which may also differ in the numerical algorithm used for the optimisation procedure. Here, we considered four different ICA algorithms, which are described in more detail in Protocol S1: the JADE (or “JointDiag”) algorithm [59], the “FastICA” algorithm [31], the “KernelICA” algorithm [32], and the “RADICAL” algorithm [33].

### Estimating the number of independent components.

The estimation of the number of sources in ICA is a hard outstanding problem. While approaches to estimating the number of sources exist, for example, the Bayesian Information Criterion (BIC) in a maximum likelihood framework [34] or using the evidence bound in a variational Bayesian approach [60–62], we decided to infer the same number of components for each algorithm. There are two reasons for this. First, because of the still relatively small sample sizes of microarray experiments, estimating the correct number of components is difficult. It has therefore been conventional to use a fixed number of components [15,16]. Second, since the aim with our work was to provide a comparison between the PCA-derived components and those derived from ICA algorithms, using the same number of components for each algorithm facilitated such a comparison.

### Feature selection.

For each component that is inferred, ICA and PCA yield a corresponding list of genes and signed weights. The ICA model is based on the premise that ICA modes selectively pick out a small percentage of genes (∼1%) that are strongly activated or repressed in response to the deregulation of a particular pathway, while the great majority of genes are unaffected. Mathematically, the distribution of inferred weights must be non-gaussian, and in the gene expression context they must be supergaussian (or leptokurtic), since most of the genes in a mode belong to a gaussian component centred at zero. Thus, to find the genes that are differentially activated, it is conventional to set a threshold, typically two or three standard deviations from the mean, and to pick out those genes whose absolute weights exceed this threshold. Although a more elegant method for determining an appropriate threshold, and which is based on measuring the deviation from normality of the weight distributions, is available [20], this method is not applicable to PCA components where deviation from normality is not a requirement. Hence, since the main aim was to provide an objective comparison of ICA with PCA, we decided to use the threshold method as this method would yield approximately the same number of features per component for PCA and ICA. To focus on the pathways that dominate an ICA mode, we used the more stringent threshold of 3 sigma on either side from the zero mean, which picks out the 0.2% of genes in the tails of the signed weight distributions. Robustness of our results to the choice of threshold was evaluated by considering less stringent thresholds of 2 and 2.5 sigma. Thus, for each inferred ICA mode or principal component, we obtained a list of selected features and associated signed weights. This resulted in a mean number of approximately 160 features (3 sigma threshold) selected per component, although this number varied significantly depending on study. Importantly though, while ICA algorithms did generally capture more features per component than PCA (as we would expect since ICA algorithms seek supergaussian components), the difference in selected feature numbers was not significant (Table S2).

### K-means clustering methods: MVG-KM and PCA-KM.

To provide an objective comparison of ICA/PCA with clustering methods, the clustering step was preceded by a feature selection step which ensured that all methods selected an approximately equal number of genes. This feature selection step was performed in two different ways. For a given cohort, genes were first ranked according to their expression variance across samples. In the most-variable-genes (MVG) method, the top 15% variable genes were then selected. In the second method, we used all the distinct genes selected through PCA using the 3 sigma threshold. Since this number is less than the total number (i.e., not distinct) of features selected from the PCA components, the remaining distinct genes were selected from the ranked MVG list. Having selected the features via one of the above methods, clustering was then performed using a robust version of k-means clustering, known as partitioning around medoids [63], where k was set to 10 in order to match the number of components inferred by ICA and PCA. Thus, PCA-KM selected the same number of total features as PCA and approximately the same number as ICA, while the threshold of 15% was chosen to ensure that MVG-KM did not select less total number of features than ICA or PCA (Table S2).

### Enrichment analysis.

For the genes selected in a ICA or PCA component or for the genes in a given cluster derived from either MVG-KM or PCA-KM, enrichment analysis evaluates whether there is statistically significant enrichment of genes from a given pathway or regulatory module. For a given study *s* and inference method *m*, let *i* denote a given inferred component (or cluster) and *p* a pathway (or regulatory module). In what follows, we also use “component” to refer to the clusters of the KM-algorithms, and also use “pathway” to refer to the regulatory modules. Let *N_S_* denote the number of genes on the array of data set *s*, and *n_sp_* denote the number of genes from pathway *p* on that same array. Similarly, let *d_smi_* denote the number of genes selected in component *i*, and *t_smi_* the number of genes from pathway *p* among the selected *d_smi_* features. Then, under the null hypothesis, where the selected genes are chosen randomly, the number *t_smi_* follows a hypergeometric distribution. Specifically, the probability distribution is

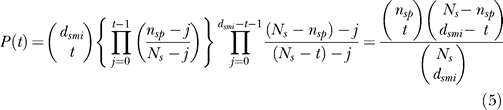
and a *p*-value can be readily computed as *P*(*t* > *t_smi_*). Note that Vandermonde's identity implies that the probability distribution is correctly normalised. Thus, for a given study and method, we can compute a *p*-value for each component-pathway pair that evaluates how enriched the component is in terms of genes from that particular pathway. To correct for multiple testing, we used the Benjamini-Hochberg procedure [64] and called a component–pathway pair association significant if the *p*-value was less than a threshold determined by setting the false discovery rate (FDR) equal to 0.05.

## Supporting Information

Figure S1 The PEI for All Breast Cancer Cohorts The pathway enrichment index, *PEI,* for all seven breast cancer cohorts in Table 1.
(2 KB PDF)

Figure S2 Vijver For the breast cancer cohort “Vijver”, we provide a heatmap of association between components/clusters and the most commonly enriched pathways and regulatory modules, as well as the association heatmap between components/clusters and breast cancer phenotypes. The strength of association between a component/cluster and a phenotype is colour-coded as follows: *p* < 10^−10^ (dark red) *p* < 0.001 (red), *p* < 0.01 (orange), *p* < 0.05 (pink), and *p* > 0.05 (white). Enriched component-pathway and component-regulatory module pairs are colour-coded in red.
(171 KB PDF)

Figure S3 Wang For the breast cancer cohort “Wang”, we provide a heatmap of association between components/clusters and the most commonly enriched pathways and regulatory modules, as well as the association heatmap between components/clusters and breast cancer phenotypes. The strength of association between a component/cluster and a phenotype is colour coded as follows: *p* < 10^−10^ (dark red) *p* < 0.001 (red), *p* < 0.01 (orange), *p* < 0.05 (pink), and *p* > 0.05 (white). Enriched component-pathway and component-regulatory module pairs are colour-coded in red.
(162 KB PDF)

Figure S4 Naderi For the breast cancer cohort “Naderi”, we provide heatmaps of association between components/clusters and the most commonly enriched pathways and regulatory modules, as well as the association heatmap between components/clusters and breast cancer phenotypes. The strength of association between a component/cluster and a phenotype is colour coded as follows: *p* < 10^−10^ (dark red) *p* < 0.001 (red), *p* < 0.01 (orange), *p* < 0.05 (pink), and *p* > 0.05 (white). Enriched component-pathway and component-regulatory module pairs are colour-coded in red.
(162 KB PDF)

Figure S5 JRH-2 For the breast cancer cohort “JRH-2”, we provide heatmaps of association between components/clusters and the most commonly enriched pathways and regulatory modules, as well as the association heatmap between components/clusters and breast cancer phenotypes. The strength of association between a component/cluster and a phenotype is colour coded as follows: *p* < 10^−10^ (dark red) *p* < 0.001 (red), *p* < 0.01 (orange), *p* < 0.05 (pink), and *p* > 0.05 (white). Enriched component-pathway and component-regulatory module pairs are colour-coded in red.
(168 KB PDF)

Figure S6 Association of Immune Response with ER Status in a Cell Line Dataset Boxplot showing the distribution of weights from an independent component enriched for immune response genes across the basal, luminal, and mesenchymal cell–line subtypes, as defined in [57]. The *p*-value of a Wilcoxon-rank sum test between the basal and luminal subtypes is given.
(3 KB PDF)

Figure S7 Association of Estrogen Signalling with ER Status (A) For each major breast cancer cohort, we give the heatmap of component expression values for a component enriched for the estrogen-signalling pathway, i.e., the heatmap matrix shown is *S_gk_A_ks_* where *k* is the component enriched for the estrogen-signalling pathway, *g* is any gene found on the array that is also in the pathway and in the selected feature set of the component, and *s* denotes the tumour sample. The ICA algorithm for which this heatmap is shown is the KernelICA algorithm. Samples have been ordered according to a k-means clustering over the set of genes. Blue denotes “upregulation”, yellow “downregulation”.
(B) For each major breast cancer cohort, we give the heatmap of expression values for the same set of genes as in (A). Thus, the heatmap matrix shown is *X_gs_* where *X_gs_* denotes the measured expression level of gene *g* in sample *s*. As before, samples have been ordered according to a k-means clustering over the represented genes.
CL, cluster labels from 2-means clustering; ER, ER status (black, ER−; grey, ER+). Red denotes relative overexpression, green underexpression.
(235 KB PDF)

Protocol S1 PCA and ICA Algorithms (98 KB PDF)

Table S1 Number of Genes per Pathway and Numbers Present on Array Platforms (26 KB TXT)

Table S2 Average Number of Selected Genes per Component/Cluster for Each Cohort and Method and Corresponding Average Number of Distinct Genes Captured by the Ten Components/Clusters (0 KB TXT)

## Abbreviations

CR: cancer related
EMT: epithelial–mesenchymal transition
ER: estrogen receptor
ICA: Independent Component Analysis
IR: immune response
IRF: interferon regulatory factor
MMP: matrix metalloproteinases
MVG: most variable genes
PCA: Principal Components Analysis
SVD: Singular Value Decomposition
