# Supplementary material for: Elucidating the Altered Transcriptional Programs in Breast Cancer using Independent Component Analysis
Source: PLoS Comput Biol. 2007 Aug 17;3(8):e161. doi: 10.1371/journal.pcbi.0030161 (PMC1950343; doi:10.1371/journal.pcbi.0030161)
Supplement: Supplementary file 1 [file pcbi.0030161.sd001.pdf]

Supplementary Information File

June 17, 2007

1 PCA and ICA algorithms

Let X be a matrix with n rows and N columns, and let X_{ij} denote the element of X in the ith row and jth column. Both ICA and PCA algorithms produce an approximate decomposition of the matrix X into the product of two matrices S and A:

$$X_{ij} = \sum_{k=1}^{K} S_{ik} A_{kj} + E_{ij}, \tag{1}$$

where $K \leq \min\{n, N\}$ is the number of components to be computed. When K is strictly smaller than $\min\{n, N\}$, it is in general impossible to pick S and A such that the error matrix E vanishes. Therefore the algorithms aim at making E as small as possible, usually in the least squares sense. This condition on E still leaves much leeway to select the matrices S and A.

The PCA method picks matrices S and A that have orthogonal columns and orthogonal rows[1], respectively, and in such a way that $\sum_{k=1}^{r} S_{ik} A_{kj}$ is the best least-squares approximation of the matrix X for all integers r in the interval $[1, K]$. Some degrees of freedom still remain, which correspond to the joint multiplication and division of the columns of S and the rows of A respectively. This defines a *scaling indeterminacy*, which can be fixed by imposing orthonormality conditions on either the columns of S or the rows of A, i.e., $\sum_{k=1}^{n} S_{ki} S_{ki} = 1$ or $\sum_{k=1}^{N} A_{ik} A_{ik} = 1$ for all i.

The ICA approach was originally dedicated to the blind source separation problem, which recovers independent source signals from linear mixtures of these [1]. ICA can be generalized to model (1) and thus, selects the matrices S and A so as to maximize the statistical independence of certain variables. Random variables are, per definition, statistically independent if their conditional probabilities are equal to the “unconditional” (i.e., marginal) probabilities. In other words, random variables are independent if the value of any one variable does not carry any information on the value of any other variable. In practice, statistical independence has to be appraised by means of finite sample sets of these random variables. In the ICA setting (1), one maximizes the statistical independence between samples sets that correspond either to the columns of S or to the rows of A, i.e., $\{S_{1i}, \ldots, S_{ni}\}_{i=1\ldots K}$ or $\{A_{i1}, \ldots, A_{iN}\}_{i=1\ldots K}$. Here we consider the case where the independence assumption is set on the columns of S. A quantitative measure of independence between samples of random

[1] A matrix $Y \in \mathbb{R}^{n\times m}$ has orthogonal columns (resp. rows) if $\sum_{k=1}^{n} Y_{ki} Y_{kj} = 0$ (resp. $\sum_{k=1}^{m} Y_{ik} Y_{jk} = 0$) for all $i \neq j$.

variables is provided by a *contrast function*. The only requirement on the contrast function is that it approaches, with probability one, to a prescribed extremum (usually zero) if and only if the random variables are statistically independent and as the number of samples n (or N) goes to infinity. This leaves many possibilities for the contrast function, which may be more or less statistically motivated. Hence, each ICA algorithm consists in the optimization of a particular contrast function. Since the independent nature of the random variables is neither altered by a scaling nor by a permutation of these variables, the ICA approach presents some inherent symmetries that have to be taken in account for an efficient optimization.

Most ICA algorithms start with a *prewhitening* step, which preprocesses the data by means of *centering* followed by PCA. The centering sets the mean of each column of S to zero, while PCA identifies an orthogonal matrix A and an orthonormal matrix S, as described above. It should be noted that orthonormality of S implies a sample covariance between the columns of S that equals zero. This is in accordance with the goal of making the columns of S "as independent as possible". The orthogonality of A, however, has to be relaxed to remove some degrees of freedom in the choice of the best decomposition of X. The ICA step per se amounts to finding the orthonormal transformation of S that optimizes the contrast between the columns of S. Thus, a contrast function that estimates the degree of statistical independence of the columns of S is needed.

A large variety of ICA algorithms have been developed during the last decade. They differ on two points: the chosen contrast function and the numerical method utilized to find its optimum. Four different ICA approaches are involved in the present work. They are briefly described next.

The JADE (or "Joint Diagnolisation") algorithm [3] constructs a set of matrices based on fourth order statistics, i.e., the cumulant matrices, which are diagonal in case of statistically independent random variables. The independent components are identified by a succession of Jacobi rotations to diagonalize simultaneously all the matrices as well as possible.

The FastICA approach [2] is motivated by the central limit theorem, which states that an infinite linear combination of random variables asymptotically converges to a gaussian variable. Thus, each combination of random variables is expected to be more gaussian than the original ones, which should be the most nongaussian. This intuition is confirmed by a property of the kurtosis of a random variable, which is a measure of its non-gaussianity. A theorem states that the kurtosis of the sum of two independent variables Y_1 and Y_2 presents a smaller absolute value than the largest absolute value of the kurtosis among these variables, i.e.,

$$|\kappa(Y_1 + Y_2)| \leq \max(|\kappa(Y_1)|, |\kappa(Y_2)|),$$

where $\kappa(Y)$ denotes the kurtosis of the random variable Y. The FastICA algorithm identifies the most nongaussian variables by maximizing a measure of nongaussianity by means of a variant to the Newton iteration. The kurtosis is a possible choice for this measure among many further heuristic extensions. In this work, we used the "pow3" approximation of the nongaussianity (equation (8.33) in [2]).

Bach and Jordan [4] propose a measure of independence based on a nonlinear generalization of the Pearson's correlation coefficient. This nonlinear measure is transformed to a

linear estimator in a higher dimensional space by means of kernels. The optimization of this contrast by gradient-descent constitutes the KernelICA algorithm.

The mutual information of random variables is a positive quantity that equals zero only in case of independent variables. It satisfies thus the requirements to be a contrast. Nevertheless, its evaluation is so complex that approximation methods are needed. The RADICAL algorithm [5] identifies an accurate and computationally efficient approximation to the mutual information based on order statistics and spacings. This contrast is optimized by means of Jacobi rotations.

Further details about ICA algorithms

Let us first fix the notations.

As previously mentioned, ICA is based on the model

$$X = SA + E,$$

where $X \in \mathbb{R}^{n \times N}$, $S \in \mathbb{R}^{n \times K}$, $A \in \mathbb{R}^{K \times N}$ and E is minimized in the least-square sense. We assume that the columns of X have mean zero. Most ICA algorithms consider the square problem, i.e., $K = \min(n, N)$. However, in many applications both n and N are large, while K, although unknown, is generally smaller. Thus, ICA algorithms generally start with a dimensional reduction step which projects the data matrix X onto a smaller matrix $\tilde{X} \in \mathbb{R}^{n \times K}$. This dimensional reduction is typically performed using a PCA on the dominant K-eigenvectors. The ICA decomposition then becomes

$$\tilde{X} = \tilde{S}\tilde{A} \tag{2}$$

where $\tilde{A}$ is now a square $K \times K$ matrix. In the literature this is usually phrased as the demixing model

$$Z = \tilde{X}W^T, \tag{3}$$

which is the inverse of (2). Thus, ICA performs a linear transformation of some observations $\tilde{X}$ into a matrix Z, the columns of which are assumed to represent samples of independent signals. Comparisons between (2) and (3) lead to the following identifications,

$$Z = \tilde{S} \qquad \text{and} \qquad W = (\tilde{A}^{-1})^T.$$

The notations related to the demixing model (3) will be used in the remainder of this section. Finally, inverting the PCA operation allows to return into the original higher dimensional space.

An ICA algorithm is basically the optimization of a particular contrast function. The contrast is a real-valued function $\gamma : \mathcal{M} \to \mathbb{R}$ defined for a matrix W of the matrix manifold $\mathcal{M}$. W is usually called the demixing matrix. Since the independent nature of random variables is neither altered by a scaling nor by a permutation of these variables, the contrast presents some inherent symmetries. Optimizing functions with symmetries is awkward unless some constraints are introduced. In case of ICA, the matrix W is usually assumed to be orthogonal, i.e., $WW^T = I$. In the remainder of this section, the contrast and the optimization process of several ICA algorithms are detailed.

1.1 Joint approximate diagonalization of a set of matrices

These methods are based on the approximate joint diagonalization of a set of matrices. Joint diagonalization of a set of m matrices $\mathcal{C} = \{C^{(i)} | i = 1, \ldots, m\}$ means that there exist a matrix W, such that all the matrices of the set $\{WC^{(i)}W^T | i = 1, \ldots, m\}$ are diagonal. It is usually impossible to identify a joint diagonalizer W that diagonalizes exactly all the matrices of $\mathcal{C}$. The problem is thus relaxed to diagonalize these matrices as well as possible, which refers to an optimization process.

Several cost functions are conceivable, but a frequently encountered one is,

$$\gamma(W) = \sum_{i}^{m} \|\mathrm{off}(WC_iW^T)\|_F^2, \tag{4}$$

where $\|\cdot\|$ denotes the Frobenius norm and $\mathrm{off}(M)$ is a matrix with entries identical to those of M, except on the diagonal, which contains only zero-valued elements. This cost function means that one wants to minimize the sum of the squares of all non-diagonal elements of the matrices of $\mathcal{C}$. This is consistent with performing the best approximate joint diagonalization of these matrices.

Several alternatives are conceivable for the matrices C_i to identify a joint diagonalizer W that performs ICA as well.

First is to consider covariance matrices. Such matrices are indeed diagonal for statistically independent variables and are affected by a linear transformation $Z = XW^T$ as follows,

$$E[Z^TZ] = WE[X^TX]W^T,$$

where $E[\cdot]$ is the expectation operator. This fits perfectly into the framework of joint diagonalization. In the same spirit, shifted covariance matrices are also diagonalizable by the demixing matrix W,

$$E[Z_{(0)}^T Z_{(k)}] = WE[X_{(0)}^T X_{(k)}]W^T,$$

where $Z_{(0)}$ denotes the signal defined by the samples $\{z_1, z_2, z_{n-k}\}$, while $Z_{(k)}$ stands for the shifted signal $\{z_k, z_{k+1}, z_n\}$.

The most frequently encountered type of matrices for performing ICA are the cumulant matrices, which are based on fourth order statistics [3]. The cumulant matrix $Q_X(M)$ associated to a N-variate zero-mean random variable X and to a $N \times N$ matrix M is defined by

$$Q_X(M) = E[(XMX^T)X^TX] - E[X^TX]\mathrm{tr}(ME[X^TX]) - E[X^TX](M + M^T)E[X^TX],$$

where $\mathrm{tr}(\cdot)$ denotes the trace.
It is possible to show that, within the ICA model $Z = XW^T$, the demixing matrix W diagonalize the cumulant matrix related to the observations X, i.e., the matrix $WQ_X(M)W^T$ is diagonal, whatever the matrix M. A set of cumulant matrices is generated by selecting matrices M as the orthogonal basis of the linear space of $N \times N$ symmetric matrices.

The famous JADE algorithm is based on contrast (4) and related to the cumulant matrices [3]. As it was previously mentioned, the ICA optimization is usually restricted to orthogonal matrices W. A classical way to achieve an optimization with such constraints is to perform Jacobi rotations. This consists in computing a planar rotation at each iteration. The Jacobi algorithm has two advantages. First is that only one parameter is varying at each iteration, such that a line search method can be performed. Then, the resulting matrix is ensured to be orthogonal since it is the product of several rotation matrices.

1.2 FastICA

The FastICA algorithm [2] is motivated by the central limit theorem, which states, informally speaking, that the sum of independent random variables converges (in distribution) to a Gaussian variable as the number of terms tends to infinity. Thus, each combination of random variables is expected to be more gaussian than the original ones, which should be the most nongaussian. This intuition is confirmed by a property of the kurtosis of a random variable, which is a measure of its non-gaussianity. A theorem states that the kurtosis of the sum of two independent variables Y_1 and Y_2 presents a smaller absolute value than the largest absolute value of the kurtosis among these variables, i.e.,

$$|\kappa(Y_1 + Y_2)| \leq \max(|\kappa(Y_1)|, |\kappa(Y_2)|),$$

where $\kappa(Y)$ denotes the kurtosis of the random variable Y. The FastICA algorithm identifies the most nongaussian variables by maximizing a measure of nongaussianity. The kurtosis is a possible choice for this measure. But usually, a heuristic approach is considered. A distance to gaussianity of the one-dimensional random variable $z = Xw^T$ can be defined by

$$\gamma(w) = (E[G(Xw^T)] - E[G(g)])^2, \tag{5}$$

where w is a vector in $\mathbb{R}^N$, g is a gaussian variable with same mean and variance as the random variable z. It is important to note that FastICA is one-unit based. Within the ICA framework $Z = XW^T$, the FastICA contrast (5) identifies the matrix W in a column-wise manner and not at once, as do most ICA algorithms. Thus, the optimization problem is

$$\max_{w \in \mathbb{R}^N} \gamma(w) \quad \text{such that} \quad ww^T = 1,$$

where the constraint on the norm of w is introduced because of the scale symmetry of the contrast. The FastICA algorithm solves that optimization problem by means of a variant to the Newton iteration. This one-unit algorithm is then used in a deflation scheme to identify different columns of the demixing matrix W.

1.3 KernelICA

This algorithm is based on a generalization of the Pearson correlation coefficient, called the $\mathcal{F}$-correlation. It is proven in [4] that two random variables z_1 and z_2 are statistically independent if and only if the $\mathcal{F}$-correlation $\rho_{\mathcal{F}}$ vanishes, $\rho_{\mathcal{F}}$ being defined by,

$$\rho_{\mathcal{F}} = \max_{f_1, f_2 \in \mathcal{F}} \operatorname{corr}(f_1(z_1) f_2(z_2)),$$

where $\mathrm{corr}(x,y)$ is the Pearson correlation coefficient between the random variables x and y and $\mathcal{F}$ is a vector space of functions from $\mathbb{R}$ to $\mathbb{R}$. This quantity seems complex to evaluate, but it can be formulated as a linear problem by means of kernel methods. $\rho_{\mathcal{F}}$ is indeed the largest eigenvalue of a generalized eigenvalue problem. This gives a convenient contrast for the two-dimensional ICA problem. Generalization to higher dimensions is rather straightforward. The ICA problem consists then in minimizing the largest eigenvalue of a (nN)-dimensional generalized eigenvalue problem, with n and N being the number of measurements and components,respectively.

The KernelICA algorithm [4] optimizes this contrast by means of a gradient-descent approach on the orthogonal group,

$$\mathcal{O}(n) = \{W \in \mathbb{R}^{N\times N} | WW^T = I\}.$$

This fits within the framework of optimization on matrix manifolds. The central idea of that theory consists in incorporating the constraints directly into the search space. This allows to perform unconstrained optimization over a nonlinear space instead of the classical constrained optimization over a linear space. Most classical unconstrained optimization methods have been generalized to the optimization over matrix manifolds. This is in particular the case of the gradient-descent method.

1.4 RADICAL

The mutual information $J(Z)$ of the multivariate random variable Z is a notion of information theory that presents all the essential characteristics to be a contrast function: it is always non negative and equals zero if and only if the variables Z are statistically independent. The mutual information is defined as the Kullback-Leibler divergence between the joint distribution and the product of the marginal distributions,

$$J(Z) = \int p(z_1,\ldots,z_N) \log \frac{p(z_1,\ldots,z_N)}{p(z_1)\ldots p(z_N)} dz_1 \ldots dz_N.$$

The mutual information can be expressed in terms of differential entropies as follows,

$$J(Z) = \sum_{i=1}^{N} H(z_i) - H(z_1,\ldots,z_N).$$

After introduction of the demixing model $Z = XW^T$, a function defined over the space of the demixing matrices is obtained,

$$J(W) = \sum_{i=1}^{N} H(XW^T e_i^T) - \log(|W|) - H(x_1,\ldots,x_N), \tag{6}$$

where e_i is the ith basis vector. The difficulty of function (6) lies in the evaluation of the differential entropies for one-dimensional variables. An efficient estimator of these quantities was derived by considering order statistics [5]. Given a one-dimensional variable z defined by its samples, the order statistics of z is the set of samples $\{z^1,\ldots,z^n\}$ rearranged in non-decreasing order, i.e., $z^1 \leq \ldots \leq z^n$. The differential entropy of a one-dimensional variable z

defined by its order statistics $\{z^1, \ldots, z^n\}$ can be estimated by

$$\hat{H}(z) = \frac{1}{n-m} \sum_{j=1}^{n-m} \log\left(\frac{n+1}{m}(z^{(j+m)} - z^{(j)})\right), \tag{7}$$

where m is typically set to $\sqrt{n}$. The RADICAL contrast is actually the function (6) where the differential entropies are evaluated with the estimator (7),

$$\gamma(W) = \sum_{i=1}^{N} \hat{H}^{(i)}(W) - \log(|W|),$$

with $\hat{H}^{(i)}(W) = \hat{H}(XW^T e_i^T)$.

The RADICAL algorithm [5] optimizes that contrast by means of Jacobi rotations. As mentioned previously, this ensures to identify an orthogonal demixing matrix. It furthermore allows to perform the optimization over one parameter at a time. This one-dimensional optimization is accomplished by exhaustive search.

References

[1] Comon P. (1994) *Independent Component Analysis: a new concept?*. Signal Process 36:287-314.

[2] Hyvarinen A., Karhunen J, Oja E (2001) *Independent Component Analysis*. Wiley.

[3] Cardoso JF. (1999) *High-order contrasts for independent component analysis*. Neural Comput 11:157-192.

[4] Bach FR, Jordan MI (2003) *Kernel independent component analysis.* Journal of Machine Learning Research 3:1-48.

[5] Learned-Miller EG, Fisher JW (2003) *ICA using spacings estimates of entropy.* Journal of Machine Learning Research 4:1271-1295.

[6] Golub GH, Van Loan CF (1996) *Matrix Computations.* John Hopkins University Press (1996).
