# Supplementary material for: Elucidating the Altered Transcriptional Programs in Breast Cancer using Independent Component Analysis
Source: PLoS Comput Biol. 2007 Aug 17;3(8):e161. doi: 10.1371/journal.pcbi.0030161 (PMC1950343; doi:10.1371/journal.pcbi.0030161)
Supplement: Supplementary file 2 [file pcbi.0030161.sg001.pdf]

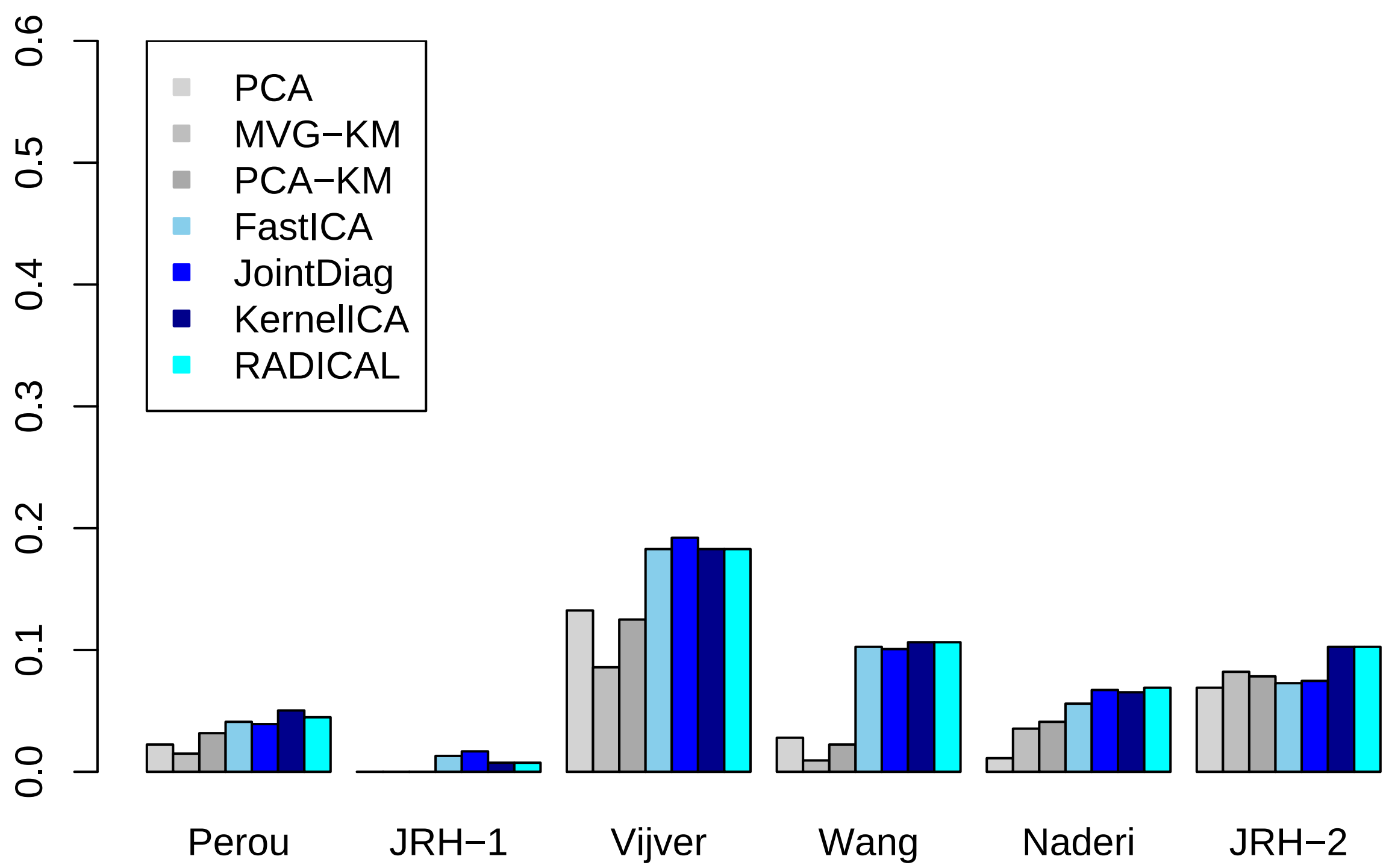
0.0
0.1
0.2
0.3
0.4
0.5
0.6
PCA
MVG−KM
PCA−KM
FastICA
JointDiag
KernelICA
RADICAL
Perou
JRH−1
Vijver
Wang
Naderi
JRH−2
