# Supplementary material for: Elucidating the Altered Transcriptional Programs in Breast Cancer using Independent Component Analysis
Source: PLoS Comput Biol. 2007 Aug 17;3(8):e161. doi: 10.1371/journal.pcbi.0030161 (PMC1950343; doi:10.1371/journal.pcbi.0030161)
Supplement: Supplementary file 4 [file pcbi.0030161.sg003.pdf]

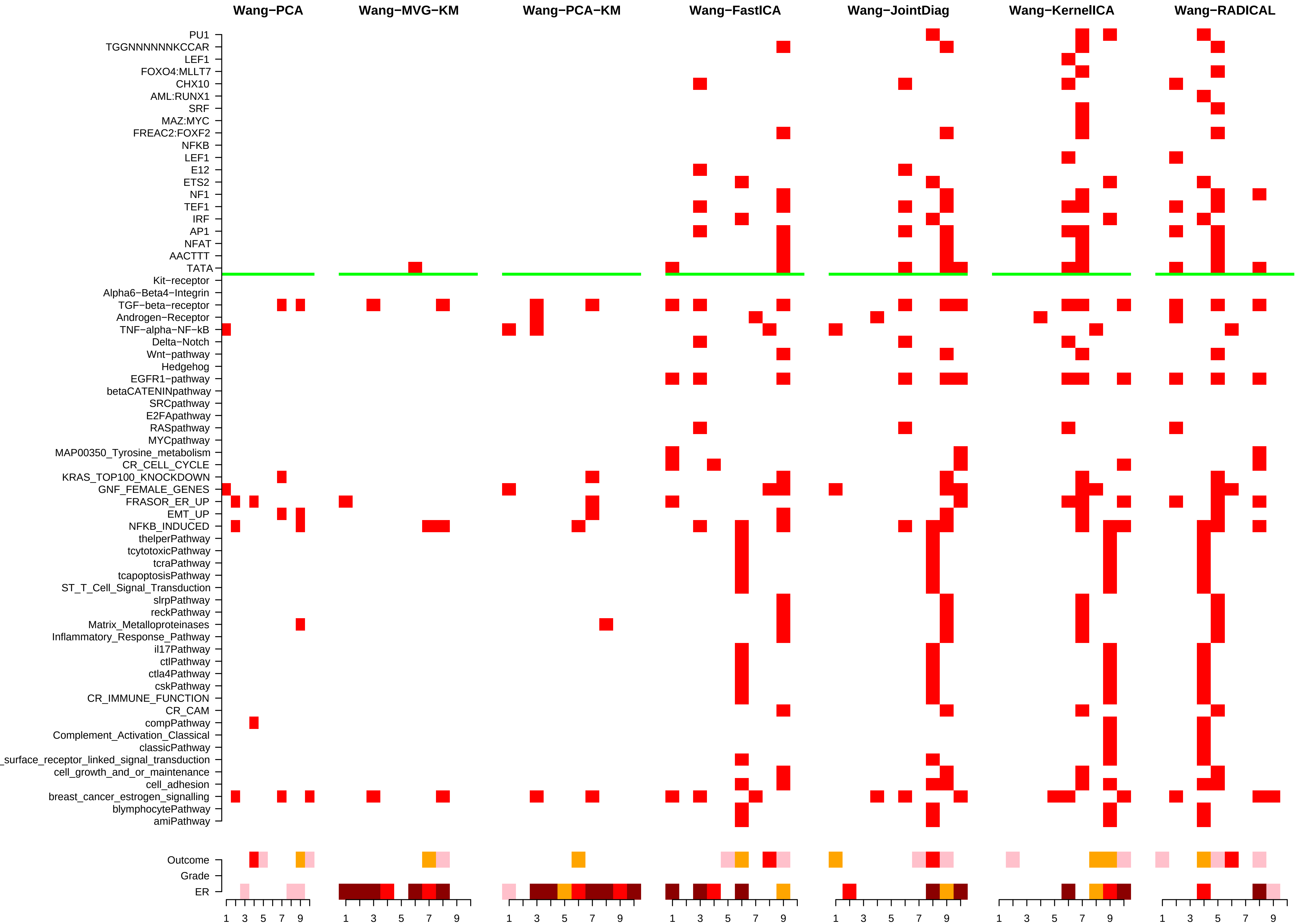

Wang−PCA
Wang−MVG−KM
Wang−PCA−KM
Wang−FastICA
Wang−JointDiag
Wang−KernelICA
Wang−RADICAL
PU1
TGGNNNNNNKCCAR
LEF1
FOXO4:MLLT7
CHX10
AML:RUNX1
SRF
MAZ:MYC
FREAC2:FOXF2
NFKB
LEF1
E12
ETS2
NF1
TEF1
IRF
AP1
NFAT
AACTTT
TATA
Kit−receptor
Alpha6−Beta4−Integrin
TGF−beta−receptor
Androgen−Receptor
TNF−alpha−NF−kB
Delta−Notch
Wnt−pathway
Hedgehog
EGFR1−pathway
betaCATENINpathway
SRCpathway
E2FApathway
RASpathway
MYCpathway
MAP00350_Tyrosine_metabolism
CR_CELL_CYCLE
KRAS_TOP100_KNOCKDOWN
GNF_FEMALE_GENES
FRASOR_ER_UP
EMT_UP
NFKB_INDUCED
thelperPathway
tcytotoxicPathway
tcraPathway
tcapoptosisPathway
ST_T_Cell_Signal_Transduction
slrpPathway
reckPathway
Matrix_Metalloproteinases
Inflammatory_Response_Pathway
il17Pathway
ctlPathway
ctla4Pathway
cskPathway
CR_IMMUNE_FUNCTION
CR_CAM
compPathway
Complement_Activation_Classical
classicPathway
_surface_receptor_linked_signal_transduction
cell_growth_and_or_maintenance
cell_adhesion
breast_cancer_estrogen_signalling
blymphocytePathway
amiPathway
Outcome
Grade
ER
1 3 5 7 9
