# Supplementary material for: Elucidating the Altered Transcriptional Programs in Breast Cancer using Independent Component Analysis
Source: PLoS Comput Biol. 2007 Aug 17;3(8):e161. doi: 10.1371/journal.pcbi.0030161 (PMC1950343; doi:10.1371/journal.pcbi.0030161)
Supplement: Supplementary file 7 [file pcbi.0030161.sg006.pdf]

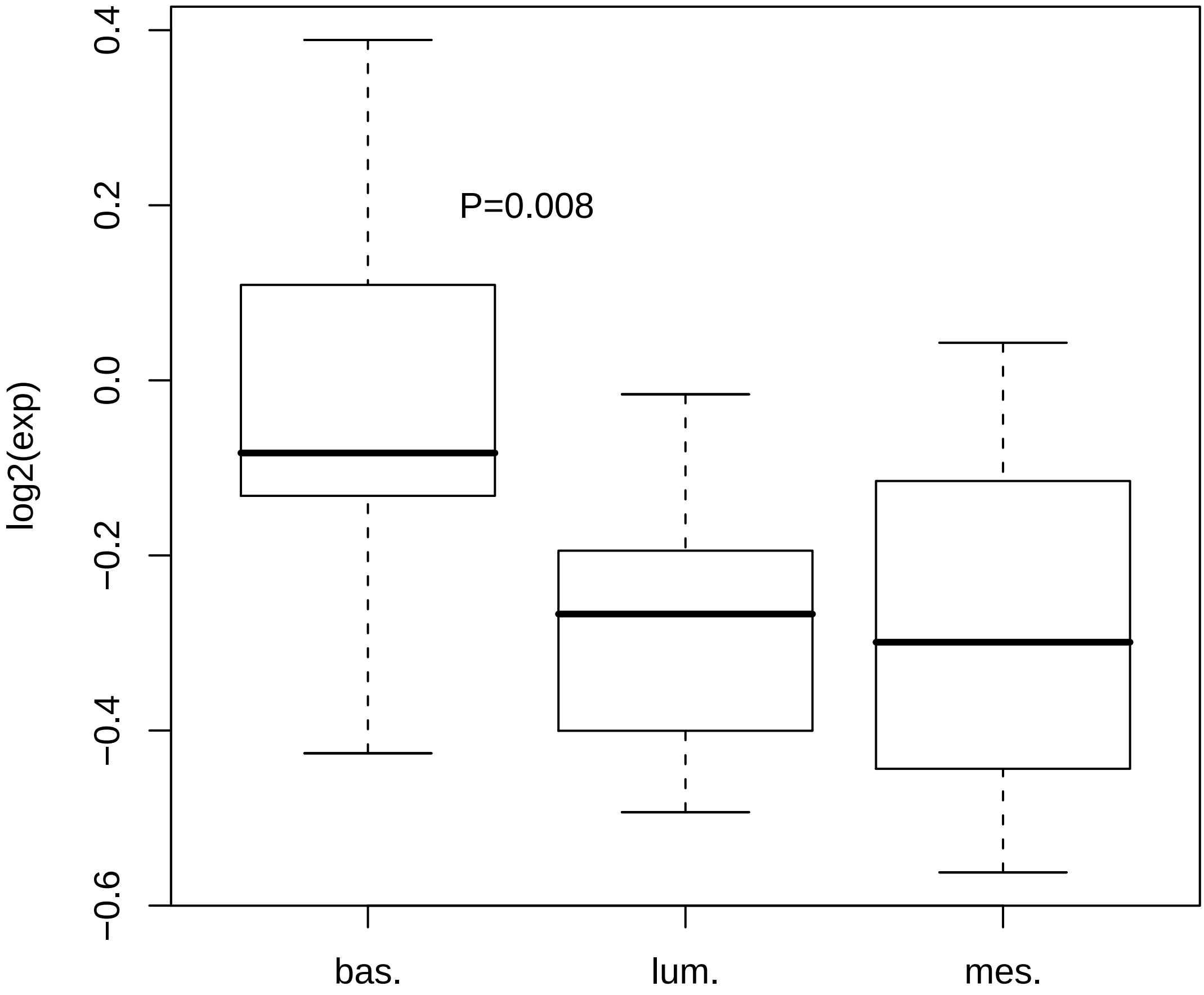

log2(exp)
0.4
0.2
0.0
−0.2
−0.4
−0.6
P=0.008
bas.
lum.
mes.
