# Supplementary material for: Elucidating the Altered Transcriptional Programs in Breast Cancer using Independent Component Analysis
Source: PLoS Comput Biol. 2007 Aug 17;3(8):e161. doi: 10.1371/journal.pcbi.0030161 (PMC1950343; doi:10.1371/journal.pcbi.0030161)
Supplement: Supplementary file 8 [file pcbi.0030161.sg007.pdf]

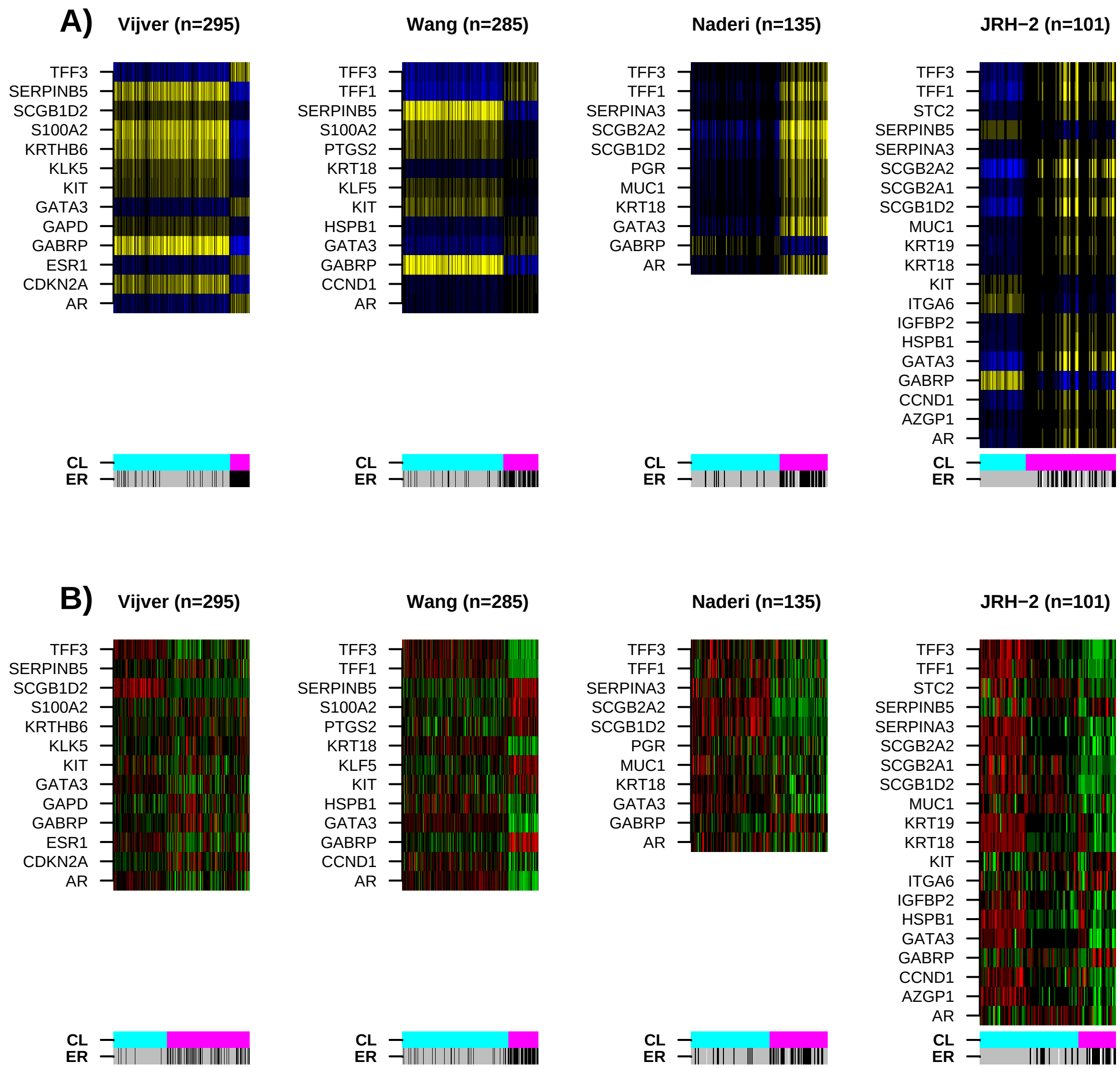

A)
Vijver (n=295)
TFF3
SERPINB5
SCGB1D2
S100A2
KRTHB6
KLK5
KIT
GATA3
GAPD
GABRP
ESR1
CDKN2A
AR
CL
ER
Wang (n=285)
TFF3
TFF1
SERPINB5
S100A2
PTGS2
KRT18
KLF5
KIT
HSPB1
GATA3
GABRP
CCND1
AR
CL
ER
Naderi (n=135)
TFF3
TFF1
SERPINA3
SCGB2A2
SCGB1D2
PGR
MUC1
KRT18
GATA3
GABRP
AR
CL
ER
JRH−2 (n=101)
TFF3
TFF1
STC2
SERPINB5
SERPINA3
SCGB2A2
SCGB2A1
SCGB1D2
MUC1
KRT19
KRT18
KIT
ITGA6
IGFBP2
HSPB1
GATA3
GABRP
CCND1
AZGP1
AR
CL
ER
B)
Vijver (n=295)
Wang (n=285)
Naderi (n=135)
JRH−2 (n=101)
